# Pacific Islanders in the Era of COVID-19: an Overlooked Community in Need

**DOI:** 10.1007/s40615-021-01075-8

**Published:** 2021-06-24

**Authors:** Leah Cha, Thomas Le, Taunuu Ve’e, Natalie T. Ah Soon, Winston Tseng

**Affiliations:** 1 University of Wisconsin School of Medicine and Public Health, Madison, WI USA; 2 Johns Hopkins University School of Medicine, Baltimore, MD USA; 3 Regional Pacific Islander Taskforce, Hayward, CA USA; 4 RAMS, Inc. (Richmond Area Multi-Services), San Francisco, CA USA; 5 Berkeley Public Health, University of California, Berkeley Berkeley Public Health, 2199 Addison St, Room 435, CA Berkeley, USA

**Keywords:** COVID-19, Epidemics, Pacific Islander, Racial disparities, Health equity

## Abstract

**Background:**

Pacific Islanders (PIs), an indigenous, diverse population in the USA, have endured generational burdens of Western colonization and institutional racism that placed this population at socioeconomic and health disadvantages, such as in poverty, chronic disease, and now COVID-19. However, little is known about the impact of COVID-19 on this historically disadvantaged population. This study assessed the extent US PIs have been adversely affected by COVID-19 across the 50 states.

**Methods:**

Using state-level national data as of September 9th, 2020, we conducted a secondary-data analysis of COVID-19 cases and deaths in PIs relative to their population representation and other racial groups, case odds ratios, and age-adjusted standard mortality ratios.

**Key Results:**

Only 46% of states reported PI cases and 36% of states reported PI deaths. Of 23 states with available data on PIs, PIs were overrepresented in COVID-19 cases and deaths relative to their population representation in 21 and 14 states, respectively. The proportion of COVID-19 cases and deaths to the PI population was highest among all racial groups in 15 and 9 states, respectively. PIs had higher odds of exposure to COVID-19 than Whites in 21 of 23 states, and higher number of observed deaths than expected in 6 of 7 states with available PI data.

**Conclusions:**

Engaging PI community-based and faith-based organizations in medical and public health outreach efforts, health workforce employment and training programs, along with granular data collection and reporting, are vital to mitigate the disproportionate effects of COVID-19 on this population.

## Background

US Pacific Islanders (PIs) represent a culturally diverse population of more than 20 ethnic subgroups with rich indigenous ties across the Pacific regions of Polynesia, Micronesia, and Melanesia [1]. Compared to most racial groups, PIs suffer from a disproportionate health burden, with lower average life expectancies and higher mortality rates. These health disparities are deeply rooted in Western colonial history, the military industrial complex, and associated social determinants in the Pacific Islands [1, 2]. Today, this complex mix of social inequities makes PIs particularly vulnerable to COVID-19 exposure and complications in the conterminous USA, Hawaii, and US-affiliated Pacific Island jurisdictions, including the territories of American Samoa, Guam, and Commonwealth of the Northern Marianas Islands as well as the countries of the Compact of Freely Associated States of Micronesia (Republic of Marshall Islands, Republic of Palau, and Federated States of Micronesia — Chuuk, Pohnpei, Kosrae, and Yap). Throughout the paper, the term “Pacific Islanders” and the federal racial classification “Native Hawaiians or other Pacific Islanders” refer to the same racial group. “Pacific Islanders” is the preferred, more inclusive term PIs self-identify as and is representative of all PI ethnic subgroups [3, 4].

The persistent health crisis facing PIs, now reaching new heights during the COVID-19 pandemic, have origins in colonial historical trauma [1, 4]. Soon after encountering Western explorers, PIs were subjected to colonial rule and subsequent lethal infectious diseases (e.g., measles, influenza), leading to catastrophic effects on these isolated indigenous populations. The 1918–1920 influenza pandemic killed substantial proportions of the total population in Western Samoa (19–22%), Tahiti (10%), Tonga (4–8%), Fiji (5%), and Guam (5%) [5]. The disease burden among PIs can also be attributed to US military and administrative control of the Pacific Islands. For instance, the US detonation of 67 thermonuclear devices in the Marshall Islands as part of the US Nuclear Weapons Testing Program led to radioactive contamination and destruction of traditional food sources and health practices, resulting in epidemics of cancer, obesity, diabetes, and cardiovascular disease [1, 6]. These epidemics serve as a sobering reminder of history repeating itself with COVID-19 devastating PIs today.

The potential for COVID-19 to have detrimental impacts on PIs is concerning and requires urgent attention. Historically, PI disparities have been diluted, as PIs have been grouped with Asian Americans in a single racial category, despite PIs and Asian Americans having dramatically different histories and cultures [1]. It was not until 1997 that the Office of Management and Budget (OMB) Directive-15 first recognized PIs as a distinct racial category and issued revised standards for reporting of the “Asian or Pacific Islander” racial classification into two distinct racial categories—“Asian” and “Native Hawaiian or other Pacific Islander [7].” In 2011, the Department of Health and Human Services (DHHS) outlined data collection guidelines to further disaggregate PIs into ethnic subgroups, such as Native Hawaiian, Chamorro, Samoan, and Other Pacific Islander [8].

These national data disaggregation standards and associated initiatives have helped to unveil numerous disease burdens among PIs that were not evident previously. However, much remains unknown about the state of PI health in the USA. PIs continue to be largely overlooked in healthcare, research, and policy considerations despite these national data guidelines. Only a small proportion of states or counties are disaggregating or reporting PI data, making it difficult to monitor the key healthcare and social disparities in this population. Additionally, numerous reports across the media and scientific communities have documented COVID-19 disparities in Black, Hispanic, and American Indian populations, but have left PIs out of national discussions [9, 10], even though PIs have been just as adversely affected. One regional study found that PIs are disproportionately affected by COVID-19 cases compared to Black and American Indian populations in select states (California, Hawaii, Oregon, Utah, and Washington) [11]. However, no studies have focused on the national impacts of COVID-19 on PIs [11, 12]. To address this gap, this study sought to assess the national burden of COVID-19 cases and deaths in PIs in comparison to other racial groups across the 50 US states.

## Methods

### Study Design

A secondary data analysis of COVID-19 state-level data across the 50 US states was conducted to assess the burden of COVID-19 cases and deaths among PIs relative to other racial groups, including American Indian or Alaska Native, Asian, Black or African American, Hispanic or Latino, and White. This study was conducted in partnership with the Pacific Islander community through the Regional Pacific Islander Task Force (RPITF), which is an institutional member of the Pacific Islander COVID-19 Response Team. This study’s findings were reviewed with the Pacific Islander community through the RPITF and their feedback was included in the final version of this manuscript.

### Data Sources

COVID-19 case and death data by state and race were obtained from the COVID-19 Tracking Project [13]. At the time of data abstraction, this dataset collected race alone data directly from websites of local or state/territory public health authorities and updates the data biweekly for public use. COVID-19 death data, stratified by state and age group were obtained from the Centers for Disease Control and Prevention (CDC) Provisional COVID-19 Death Counts by Sex, Age, and State, available on the CDC website [14]. As the CDC dataset censored death counts less than 10, there were no age-stratified data for some states. Both datasets were abstracted as of September 9th, 2020. State-level population data by race and age were taken from the 2018 American Community Survey (ACS) 1-year estimates [15]. The ACS dataset represents race alone data (e.g., Native Hawaiian or other Pacific Islander alone), consistent with national data standards for assessing COVID-19 rates by race.

All datasets included the following six racial categories: American Indian or Alaska Native, Asian, Black or African American, Hispanic or Latino, Native Hawaiian or other Pacific Islander, and White. We excluded US states/territories where PI data were not available, not reported, or insufficient. Of the US territories, Guam and Northern Mariana Islands report overall COVID-19 cases and deaths, and only Guam reports overall COVID-19 cases for PIs. Data on US territories were excluded from the analysis given insufficient COVID-19 data on PIs. Insufficient data included absence of COVID-19 data on PI cases or deaths, or absence of PI population data as an aggregate or by age strata.

### Analysis

The proportions of COVID-19 cases and deaths in six racial groups were calculated for each state that had publicly available PI data. The denominator used for these calculations excluded unknown race. We also calculated the proportion of cases and deaths relative to the population representation for each racial group by state. Given small counts of Pacific Islanders relative to the total US population, the proportion of cases or deaths to the population was rounded to the hundredth decimal.

An odds ratio (OR) and 95% confidence interval (CI) of case rates were calculated comparing the odds of COVID-19 cases in PIs with that of Whites for each state [16]. ORs and 95% CIs were also calculated comparing the odds of COVID-19-related deaths in PIs with that of Whites for each state. Odds of COVID-19-related deaths among COVID-19 cases were calculated for each racial group by state. Age was not adjusted as the datasets used in this study do not have age-stratified racial data.

In order to account for the lack of mortality data on racial groups stratified by age, standardized mortality ratios (SMR), which compared observed and expected death counts, were computed and indirectly adjusted for age. Expected death for COVID-19 for each racial group within a given state was calculated by: (1) counting the number of COVID-19 deaths within 10-year age strata, and (2) dividing that number by the total population within that age strata for each state. These age-stratified mortality rates were then applied to the racial population within each age strata and summed to calculate an expected death estimate from COVID-19. The expected death estimate was compared against the actual COVID-19 death estimate to calculate SMR. 95% CI was calculated from the SMR using a Mid-P test based on Miettinen’s modification [17].

All analyses were conducted using Microsoft Excel (Microsoft, Redmond, WA).

## Results

### COVID-19 Cases and Deaths in PIs

As of September 9th, 2020, only 46% of states reported cases and 36% of states reported deaths for PIs. Overall, 54% of states did not report any COVID-19 data on PIs (cases or deaths), 16% of states aggregated them with Asian Americans, and 38% of states reported them in an “other” category due to small counts.

Among 23 states with available COVID-19 data on PI cases, PIs represented 0.35% of the total population (485,898) and made up 0.39% of cases (15,183 cases). Among 18 states with COVID-19 data on PI deaths, PIs represented 0.26% of the total population (331,659) and 0.13% of deaths (221 deaths). The number of COVID-19 cases and deaths in PIs was highest in California (2778 cases; 59 deaths) and Arkansas (2374 cases; 46 deaths) (Table 1). Arkansas had the highest proportion of PI COVID-19 cases (12.55%) and deaths (16.03%) by racial population representation across all states. In Arkansas, the proportion of COVID-19 cases in PIs were 16 times higher than Whites and 8 times higher than Blacks. The proportion of COVID-19 deaths in PIs in Arkansas were 19 times higher than Whites and 11 times higher than Blacks. Furthermore, PIs were overrepresented in COVID-19 cases compared to their population representation in 21 states, including Arkansas (13 times), Iowa (11 times), Louisiana (7 times), Illinois (6 times), and Alaska (6 times) (Table 2). The proportion of COVID-19 PI deaths was greater than their population representation in 14 states, including Arkansas (16 times), Alaska (12 times), Iowa (9 times), Utah (6 times), and Louisiana (6 times) (Table 3). However, the numbers of COVID-19 cases or deaths for PIs were likely underreported. Fifty-seven percent of PIs report multi-racial descent (842,185), but our data sources only report “PI race alone,” and not “PI race alone or in combination with other races.”

**Table 1:** Population representation of Pacific Islanders (PI) and COVID-19 cases and deaths by state

States^e^	No. (%)^b^					
	Population^a^		Cases^c^		Deaths^d^	
	PI	White	PI	White	PI	White
California	155,739 (0.39)	23,535,388 (59.50)	2778 (0.55)	84,646 (16.89)	59 (0.43)	4019 (29.59)
Arkansas	9398 (0.31)	2,306,096 (76.52)	2374 (3.89)	37,400 (61.21)	46 (4.97)	594 (64.15)
Utah	29,362 (0.93)	2,708,195 (85.67)	2318 (4.64)	24,347 (48.77)	23 (5.67)	219 (53.94)
Hawaii	144,971 (10.21)	345,652 (24.33)	1830 (43.14)	664 (15.65)	–	–
Washington	53,924 (0.72)	5,633,263 (74.76)	1455 (2.69)	20,520 (37.89)	32 (1.64)	1314 (67.28)
Iowa	3463 (0.11)	2,846,099 (90.18)	695 (1.20)	47,981 (83.14)	11 (1.03)	979 (91.41)
Oregon	18,758 (0.45)	3,514,983 (83.88)	538 (2.15)	11,468 (45.84)	7 (1.65)	319 (75.24)
Illinois	5317 (0.04)	9,135,145 (71.70)	479 (0.25)	70,474 (36.90)	9 (0.11)	3809 (47.14)
Georgia	14,049 (0.13)	6,127,645 (58.25)	308 (0.15)	81,379 (39.95)	9 (0.15)	2965 (48.88)
North Carolina	10,218 (0.10)	7,098,569 (68.36)	273 (0.20)	79,184 (57.08)	4 (0.14)	1674 (59.49)
Colorado	7585 (0.13)	4,790,677 (84.11)	239 (0.47)	22,212 (43.65)	5 (0.27)	1179 (63.80)
Alaska	7958 (1.08)	474,555 (64.35)	235 (6.58)	1549 (43.37)	5 (12.50)	18 (45.00)
Ohio	4228 (0.04)	9,470,940 (81.02)	185 (0.16)	71,981 (61.38)	1 (0.02)	3352 (78.24)
Louisiana	1133 (0.02)	2,877,115 (61.74)	184 (0.13)	58,538 (42.50)	6 (0.12)	2554 (51.59)
Minnesota	2396 (0.04)	4,629,375 (82.50)	125 (0.15)	36,872 (45.04)	2 (0.11)	1443 (77.21)
Tennessee	5494 (0.08)	5,235,588 (77.34)	124 (0.09)	86,311 (60.94)	2 (0.10)	1234 (64.57)
Idaho	2763 (0.16)	1,576,846 (89.89)	92 (0.41)	16,518 (73.53)	–	387 (96.27)
Nebraska	1289 (0.07)	1,666,990 (86.41)	57 (0.20)	23,200 (79.38)	0 (0.00)	319 (82.01)
Kentucky	4943 (0.11)	3,875,023 (86.72)	55 (0.15)	29,485 (80.24)	–	776 (83.80)
Wyoming	1111 (0.19)	528,453 (91.47)	14 (0.42)	2164 (64.77)	–	17 (48.57)
West Virginia	451 (0.03)	1,679,773 (93.02)	2 (0.02)	9461 (80.12)	–	58 (95.08)
Maine	465 (0.03)	1,261,453 (94.25)	2 (0.05)	3067 (71.41)	0 (0.00)	119 (96.75)
Rhode Island	883 (0.08)	852,805 (80.66)	0 (0.00)	6326 (36.99)	0 (0.00)	701 (80.57)
Guam^f^	–	–	783 (48.60)	239 (14.84)	–	–

^a^Population data represent Native Hawaiian or other Pacific Islander alone from the 2018 American Community Survey 1-year estimates. COVID-19 data are adapted from https://covidtracking.com/race and last updated on September 9th, 2020. The numbers of COVID-19 cases or deaths are likely underreported as Native Hawaiian or other Pacific Islander alone data was collected, instead of Native Hawaiian or other Pacific Islander alone or in combination with other races. Additionally, accurate reporting of states with a greater proportion of COVID-19 cases or deaths is complicated by Utah and Wyoming reporting non-mutually exclusive categories for race data

^b^The denominator used to calculate percentage of cases or deaths excludes unknown race. Given small counts of Pacific Islanders relative to the total US population, the proportion of cases or deaths to the population presented in the narrative was calculated using percentages rounded to the hundredth decimal

^c^Twenty-seven states did not report COVID-19 cases for Pacific Islanders: Alabama, Arizona, Connecticut, Delaware, Florida, Indiana, Kansas, Maryland, Massachusetts, Michigan, Mississippi, Missouri, Montana, Nevada, New Hampshire, New Jersey, New Mexico, New York, North Dakota, Oklahoma, Pennsylvania, South Carolina, South Dakota, Texas, Vermont, Virginia, and Wisconsin

^d^Thirty-two states did not report COVID-19 deaths for Pacific Islanders: Alabama, Arizona, Connecticut, Delaware, Florida, Hawaii, Idaho, Indiana, Kansas, Kentucky, Maryland, Massachusetts, Michigan, Mississippi, Missouri, Montana, Nevada, New Hampshire, New Jersey, New Mexico, New York, North Dakota, Oklahoma, Pennsylvania, South Carolina, South Dakota, Texas, Vermont, Virginia, West Virginia, Wisconsin, and Wyoming

^e^Eight states report COVID-19 cases or deaths for Pacific Islanders and Asians as a single panracial category: Arizona, Connecticut, Delaware, Michigan, New York, Oklahoma, Virginia, and Wisconsin

^f^Guam and Northern Marianas Islands report overall COVID-19 cases and deaths. Only Guam reports COVID-19 cases for Pacific Islanders. COVID-19 data for American Samoa are not reported. However, there are likely no COVID-19 cases and deaths in American Samoa due to early travel restrictions to and from the island. Population data for Guam was not available in the 2018 American Community Survey 1-year estimates

**Table 2:** Proportions of COVID-19 cases compared to population representation in Pacific Islanders and other racial groups

States	No.					
	Cases					
	PI	Asian	White	Black	Hispanic	AIAN
California	1.41	0.37	0.28	0.74	1.54	0.32
Arkansas	12.55	0.89	0.80	1.59	–	0.49
Utah	4.99	1.08	0.57	1.95	2.77	1.99
Hawaii	4.23	0.94	0.64	0.85	–	–
Washington	3.74	0.55	0.51	1.52	3.28	1.33
Iowa	10.91	1.91	0.92	1.98	–	3.33
Oregon	4.78	0.72	0.55	1.99	–	2.40
Illinois	6.25	0.55	0.51	1.36	2.05	0.61
Georgia	1.15	0.50	0.69	1.14	1.85	0.24
North Carolina	2.00	0.65	0.83	1.10	–	1.35
Colorado	3.62	0.74	0.52	1.32	2.07	0.68
Alaska	6.09	0.70	0.67	1.88	–	1.83
Ohio	4.00	1.22	0.76	1.97	–	0.68
Louisiana	6.50	3.36	0.69	1.28	–	0.53
Minnesota	3.75	1.14	0.55	2.92	–	0.73
Tennessee	1.13	0.60	0.79	1.33	–	0.57
Idaho	2.56	0.71	0.82	2.17	–	1.63
Nebraska	2.86	2.16	0.92	1.59	–	1.32
Kentucky	1.36	0.96	0.93	1.52	–	2.00
Wyoming	2.21	0.83	0.71	1.81	1.67	4.96
West Virginia	1.00	–	0.86	1.78	–	–
Maine	1.67	2.21	0.76	15.51	–	0.33
Rhode Island	0.00	0.59	0.46	1.86	2.87	1.00

Population data represent Native Hawaiian or other Pacific Islander alone from the 2018 American Community Survey 1-year estimates. COVID-19 data are adapted from https://covidtracking.com/race and last updated on September 9th, 2020. The numbers of COVID-19 cases or deaths are likely underreported as Native Hawaiian or other Pacific Islander alone data was collected, instead of Native Hawaiian or other Pacific Islander alone or in combination with other races. Additionally, accurate reporting of states with a greater proportion of COVID-19 cases or deaths is complicated by Utah and Wyoming reporting non-mutually exclusive categories for race data. The denominator used to calculate percentage of cases or deaths excludes unknown race. Given small counts of Pacific Islanders relative to the total US population, the proportion of cases or deaths to the population was calculated using percentages rounded to the hundredth decimal

**Table 3:** Proportions of COVID-19 deaths compared to population representation in Pacific Islanders and other racial groups

States	No.					
	Deaths					
	PI	Asian	White	Black	Hispanic	AIAN
California	1.10	0.79	0.50	1.34	1.23	0.39
Arkansas	16.03	0.75	0.84	1.51	–	0.46
Utah	6.10	2.36	0.63	1.34	1.68	7.61
Hawaii	–	–	–	–	–	–
Washington	2.28	0.85	0.90	0.86	1.12	2.06
Iowa	9.36	0.92	1.01	1.10	–	1.31
Oregon	3.67	0.98	0.90	1.44	–	2.02
Illinois	2.75	0.82	0.66	1.91	1.18	0.54
Georgia	1.15	0.42	0.84	1.35	0.64	0.24
North Carolina	1.40	0.42	0.87	1.44	–	1.16
Colorado	2.08	1.01	0.76	1.54	1.09	0.72
Alaska	11.57	1.19	0.70	0.00	–	2.32
Ohio	0.50	0.44	0.97	1.51	–	0.32
Louisiana	6.00	0.42	0.84	1.45	–	0.15
Minnesota	2.75	0.95	0.94	1.49	–	1.72
Tennessee	1.25	0.53	0.83	1.74	–	0.75
Idaho	–	0.32	1.07	1.45	–	0.76
Nebraska	0.00	1.95	0.95	1.58	–	2.95
Kentucky	–	0.82	0.97	1.62	–	0.00
Wyoming	–	0.00	0.53	4.54	0.57	15.31
West Virginia	–	–	1.02	1.29	–	–
Maine	0.00	0.00	1.03	2.29	–	0.00
Rhode Island	0.00	0.40	1.00	0.93	0.74	0.27

Population data represent Native Hawaiian or other Pacific Islander alone from the 2018 American Community Survey 1-year estimates. COVID-19 data are adapted from https://covidtracking.com/race and last updated on September 9th, 2020. The numbers of COVID-19 cases or deaths are likely underreported as Native Hawaiian or other Pacific Islander alone data was collected, instead of Native Hawaiian or other Pacific Islander alone or in combination with other races. Additionally, accurate reporting of states with a greater proportion of COVID-19 cases or deaths is complicated by Utah and Wyoming reporting non-mutually exclusive categories for race data. The denominator used to calculate percentage of cases or deaths excludes unknown race. Given small counts of Pacific Islanders relative to the total US population, the proportion of cases or deaths to the population was calculated using percentages rounded to the hundredth decimal

### COVID-19 Cases and Deaths in PIs Compared to Other Racial Groups

PIs were more likely to be exposed to COVID-19 than Whites in 21 of 23 states that had available COVID-19 case data on PIs (Table 4). The proportion of COVID-19 PI cases and deaths to the total population was also the highest across all racial groups, followed by Black or Hispanic populations, in 15 states for cases and 9 states for deaths, respectively.

**Table 4:** Odds ratios (OR) of COVID-19 cases among Pacific Islanders in comparison to other races

States	OR (95% CI)				
	PI	Asian	Black	Hispanic	AIAN
California	5.03 (4.84–5.23)	1.31 (1.29–1.32)	2.62 (2.59–2.66)	5.51 (5.47–5.56)	1.15 (1.08–1.21)
Arkansas	20.50 (19.58–21.47)	1.11 (1.04–1.19)	2.02 (1.98–2.06)	3.87 (3.79–3.95)	0.61 (0.53–0.70)
Utah	9.45 (9.04–9.87)	1.91 (1.80–2.02)	3.51 (3.31–3.72)	5.05 (4.95–5.14)	3.59 (3.38–3.82)
Hawaii	6.64 (6.08–7.26)	1.46 (1.33–1.60)	1.32 (1.04–1.69)	–	–
Washington	7.59 (7.19–8.00)	1.09 (1.05–1.14)	3.02 (2.91–3.14)	6.61 (6.48–6.73)	2.62 (2.45–2.80)
Iowa	14.64 (13.49–15.89)	2.10 (2.02–2.18)	2.19 (2.12–2.26)	3.36 (3.29–3.43)	3.75 (3.47–4.05)
Oregon	9.02 (8.26–9.85)	1.32 (1.23–1.41)	3.69 (3.45–3.94)	6.42 (6.25–6.59)	4.45 (4.12–4.80)
Illinois	12.73 (11.59–13.99)	1.07 (1.04–1.09)	2.68 (2.65–2.72)	4.08 (4.03–4.12)	1.19 (1.07–1.33)
Georgia	1.67 (1.49–1.86)	0.73 (0.70–0.75)	1.67 (1.66–1.69)	2.76 (2.73–2.80)	0.36 (0.31–0.42)
North Carolina	2.43 (2.16–2.74)	0.77 (0.74–0.80)	1.32 (1.31–1.34)	3.95 (3.90–4.00)	1.63 (1.56–1.70)
Colorado	6.98 (6.14–7.95)	1.43 (1.35–1.51)	2.55 (2.45–2.66)	4.04 (3.97–4.12)	1.31 (1.18–1.46)
Alaska	9.29 (8.09–10.68)	1.04 (0.89–1.23)	2.81 (2.45–3.23)	2.18 (1.95–2.44)	2.74 (2.53–2.96)
Ohio	5.97 (5.16–6.92)	1.62 (1.57–1.68)	2.63 (2.60–2.67)	2.41 (2.35–2.46)	0.88 (0.76–1.02)
Louisiana	9.34 (7.99–10.91)	5.32 (5.19–5.45)	1.89 (1.87–1.91)	–	0.77 (0.70–0.85)
Minnesota	6.86 (5.73–8.21)	2.10 (2.03–2.17)	5.54 (5.44– 5.65)	5.81 (5.70–5.93)	1.35 (1.25–1.46)
Tennessee	1.38 (1.15–1.65)	0.75 (0.72–0.79)	1.71 (1.68–1.73)	4.04 (3.98–4.10)	0.70 (0.61–0.80)
Idaho	3.25 (2.64–4.01)	0.86 (0.76–0.98)	2.71 (2.43–3.03)	2.70 (2.62–2.78)	2.01 (1.83–2.20)
Nebraska	3.28 (2.51–4.28)	2.40 (2.28–2.53)	1.75 (1.68–1.83)	3.94 (3.85–4.03)	1.44 (1.30–1.60)
Kentucky	1.47 (1.12–1.91)	1.04 (0.95–1.13)	1.65 (1.60–1.70)	3.41 (3.30–3.52)	2.19 (1.85–2.59)
Wyoming	3.10 (1.83–5.27)	1.18 (0.81–1.71)	2.55 (1.84–3.52)	2.37 (2.16–2.60)	7.18 (6.49–7.94)
West Virginia	0.79 (0.20–3.15)	–	2.07 (1.93–2.23)	–	–
Maine	1.77 (0.44–7.11)	2.92 (2.42–3.54)	21.55 (20.01–23.22)	3.55 (3.06–4.10)	0.43 (0.22–0.83)
Rhode Island	0.00	1.28 (1.15–1.43)	4.15 (3.94–4.36)	6.51 (6.29–6.73)	2.19 (1.73–2.78)

Case odds ratios (OR) were highest among PIs across all racial groups in Arkansas (OR 20.50, 95% CI 19.58–21.47), Iowa (14.64, 13.49–15.89), Illinois, (12.73, 11.59–13.99), Utah (9.45, 9.04–9.87), and Oregon (9.02, 8.26–9.85). Of the 15 states with COVID-19 PI death data, 6 states (40%) had death ORs that showed a protective effect of PI race compared to Whites, while 9 states (60%) showed no statistically significant effect (Table 5). The greatest protective effect of PI race was seen in Ohio (0.11, 0.02–0.79), Washington (0.33, 0.23–0.47), and Illinois (0.34, 0.17–0.65). Similar trends in terms of protective effect and non-significance for death ORs were seen across all racial groups.

**Table 5:** Odds ratios of COVID-19 deaths among Pacific Islanders in comparison to other races

States	OR (95% CI)				
	PI	Asian	Black	Hispanic	AIAN
California	0.44 (0.34–0.56)	1.24 (1.16–1.31)	1.03 (0.96–1.11)	0.45 (0.43–0.46)	0.68 (0.50–0.93)
Arkansas	1.22 (0.90–1.66)	0.81 (0.44–1.47)	0.90 (0.77–1.06)	0.33 (0.26–0.43)	0.90 (0.29–2.83)
Utah	1.10 (0.72–1.70)	2.00 (1.29–3.08)	0.62 (0.29–1.32)	0.54 (0.43–0.69)	3.53 (2.45–5.09)
Washington	0.33 (0.23–0.47)	0.86 (0.72–1.02)	0.30 (0.24–0.39)	0.18 (0.16–0.21)	0.87 (0.66–1.16)
Iowa	0.77 (0.42–1.41)	0.44 (0.29–0.65)	0.50 (0.37–0.68)	0.32 (0.25–0.40)	0.35 (0.14–0.84)
Oregon	0.46 (0.22–0.98)	0.83 (0.52–1.33)	0.43 (0.24–0.78)	0.20 (0.15–0.26)	0.50 (0.27–0.95)
Illinois	0.34 (0.17–0.65)	1.18 (1.05–1.31)	1.10 (1.04–1.16)	0.44 (0.41–0.46)	0.66 (0.37–1.18)
Georgia	0.80 (0.41–1.55)	0.69 (0.57–0.84)	0.97 (0.92–1.02)	0.27 (0.25–0.31)	0.82 (0.34–2.00)
North Carolina	0.69 (0.26–1.85)	0.62 (0.44–0.87)	1.27 (1.16–1.37)	0.30 (0.26–0.34)	0.82 (0.59–1.14)
Colorado	0.38 (0.16–0.93)	0.93 (0.71–1.21)	0.79 (0.66–0.96)	0.35 (0.31–0.39)	0.72 (0.41–1.26)
Alaska	1.85 (0.68–5.03)	1.65 (0.48–5.65)	0.00	0.23 (0.03–1.70)	1.22 (0.60–2.47)
Ohio	0.11 (0.02–0.79)	0.27 (0.20–0.37)	0.59 (0.55–0.64)	0.24 (0.19–0.29)	0.36 (0.12–1.14)
Louisiana	0.74 (0.33–1.67)	0.10 (0.07–0.14)	0.93 (0.88–0.99)	–	0.22 (0.08–0.59)
Minnesota	0.40 (0.10–1.62)	0.48 (0.39–0.60)	0.29 (0.25–0.34)	0.15 (0.12–0.19)	1.39 (0.98–1.96)
Tennessee	1.13 (0.28–4.58)	0.85 (0.53–1.35)	1.24 (1.12–1.37)	0.38 (0.32–0.45)	1.27 (0.47–3.41)

Seven states had data available on PI deaths to calculate an SMR representing COVID-19 mortality in this population and indirectly adjust for age (Table 6). A disproportionately higher number of deaths was observed than expected in PIs in 6 of 7 states, including Arkansas (84.71, 62.74–112.01), Oregon (14.20, 6.21–28.09), and Utah (10.44, 6.78–15.42).

**Table 6:** Standard mortality rates in COVID-19 among Pacific Islanders in comparison to other races

Races		California	Arkansas	Utah	Washing-ton	Oregon	North Carolina	Colorado
PI	Expected	41.91	0.54	2.20	4.61	0.49	0.96	2.77
	SMR	1.41	84.71	10.44	6.94	14.20	4.19	1.81
	95% CI	1.08–1.80	62.74–112.01	6.78–15.42	4.83–9.69	6.21–28.09	1.33–10.10	0.66–4.01
Asian	Expected	2149.00	4.96	7.64	119.34	10.92	30.87	40.36
	SMR	0.74	2.22	3.01	1.22	1.74	1.13	1.46
	95% CI	0.70–0.78	1.17–3.85	1.95–4.45	1.04–1.43	1.08–2.67	0.80–1.56	1.12–1.87
White	Expected	9160.65	655.24	370.48	1524.08	361.03	1657.48	1721.03
	SMR	0.44	0.91	0.59	0.86	0.88	1.01	0.69
	95% CI	0.43–0.45	0.84–0.98	0.52–0.67	0.82–0.91	0.79–0.98	0.96–1.06	0.65–0.73
Black	Expected	713.63	77.16	2.08	37.30	3.71	351.77	49.67
	SMR	1.47	2.75	3.36	1.74	3.23	2.47	2.40
	95% CI	1.38–1.56	2.40–3.14	1.47–6.65	1.36–2.21	1.75–5.49	2.31–2.64	1.99–2.86
His-panic	Expected	3044.55	18.01	25.52	63.85	15.71	59.15	204.77
	SMR	2.16	4.11	3.80	4.42	4.07	4.58	2.13
	95% CI	2.11–2.21	3.25–5.13	3.10–4.62	3.92–4.96	3.16–5.17	4.06–5.15	1.94–2.34
AIAN	Expected	86.86	2.90	2.76	15.02	3.37	18.05	13.46
	SMR	0.47	1.03	12.32	3.46	2.97	2.10	0.97
	95% CI	0.34–0.63	0.26–2.81	8.67–17.03	2.61–4.50	1.51–5.29	1.51–2.86	0.54–1.61

## Discussion

The adverse impact of COVID-19 on PIs can no longer be overlooked by medical, public health, and policy stakeholders. Only a few studies have documented the disproportionate burden of COVID-19 cases and deaths in PIs compared to other racial groups, and have focused on select Western US states [11, 12]. This study is the first to examine the national burden of COVID-19 cases and deaths in PIs across all US states.

We found that PIs are grossly underrepresented in data collection and reporting of COVID-19, which limit the ability to fully understand the national impact of COVID-19 on this population. PIs across the USA were overrepresented in COVID-19 cases and deaths relative to their population representation and experienced a disproportionately higher burden of COVID-19 cases and deaths than other racial groups, including Black and Hispanic populations, in the states with available PI data. We observed higher odds of COVID-19 cases in PIs compared to Whites in 21 of 23 US states. While we also found a lower odds of mortality among PIs and other racial groups compared to Whites, these calculations did not adjust for the relatively younger age population in PIs [18], which had been done for Black and Hispanic populations in the literature [19]. Using an indirect age adjustment, we observed a higher number of deaths in PIs than expected in 6 of 7 US states with available PI data. Our findings are similar to and add to the limited literature on PIs, which observed higher COVID-19 cases in PIs than Black and American Indian populations across several US states [11, 20–22]. While there have been reports demonstrating overrepresentation of racial groups in COVID-19 mortality, such as Black, Hispanic, and American Indian populations, this is the first study to our knowledge to highlight disproportionality of mortality in PIs [19, 23, 24].

The disproportionate burden of chronic illness in PIs, such as diabetes, cardiovascular disease, and cancer, may explain in part why PIs are overrepresented in COVID-19 deaths. Previous reports have shown a strong link between underlying chronic medical conditions and risk for severe illness and death from COVID-19 [11]. Nationally, PIs (51.7%; 19.8%) were 1.8 times more likely to be obese, and 2.5 times more likely to be diagnosed with diabetes than non-Hispanic Whites (NHWs) (28.7%; 8.0%) respectively [25]. In Hawaii, Native Hawaiians (404.8) were three times more likely to die from all types of cancer than NHWs (136.5). The uninsurance rate in PIs (8.3%) was also 1.4 times higher compared to NHWs (5.9%). The disease burden and lack of access to care among PIs also have roots in historical events and US military presence across the Pacific. During the Cold War, the US Nuclear Weapons Testing Program exposed Marshall Islands residents to approximately 7200 Hiroshima-size explosions, significantly altering their means of sustaining island life [6]. Beginning in the 1980s, most Micronesians migrated to the USA following the signing of treaties called the Compacts of Free Association (COFA) between the USA and three Micronesian nations (the Federated States of Micronesia, the Republic of Palau, and the Republic of the Marshall Islands). These compacts gave the US exclusive military control over these nations and the responsibility to develop their health, educational, and economic infrastructures [26]. Despite US responsibilities under these compacts, the Personal Responsibility and Work Opportunity Act of 1996 deemed COFA populations ineligible for most federal aid programs, including Medicaid. Both traumatic events effectively pushed many PIs from their homelands to other US territories and the conterminous USA.

Furthermore, these alarming COVID-19 rates may be attributed to social determinants that make PIs vulnerable to the disproportionate spread of COVID-19 [11]. PIs have been vital to COVID-19 response and recovery in the USA. A large proportion of PIs is made up of essential workers, with heavy representation in the military, security, service, and healthcare industries, increasing their risk of exposure to COVID-19. Many of these jobs, particularly those in the service industry, often do not provide livable wages or adequate personal protective equipment. PIs have also resided and worked in industries where COVID-19 resurgence and clusters were identified, particularly in states that re-opened in the first 2 weeks of May 2020, namely Arkansas, Idaho, Iowa, and Utah [22]. Moreover, indigenous PIs are a highly communal culture with a strong microsystem of social support, particularly through faith-based organizations. Many generations of families tend to live in the same household or near each other, which makes quarantining unfeasible for those residing in single dwelling homes [27]. Additionally, PIs represent incarcerated and homeless populations at disproportionate rates similar to Black and Hispanic populations, putting them at greater risk of contracting COVID-19 [28–30]. Finally, health communication and outreach critical to reducing the spread and severity of COVID-19 may have been complicated by barriers to access, including lack of health insurance, high cost burden, limited English proficiency, and unfamiliarity with the health care system [1].

Our study is limited by the lack of COVID-19 federal- and state-level data collection and reporting of PIs. This lack of data presents a challenge in understanding the depth of the pandemic’s impact on this population. Our study found that the plurality of states did not report COVID-19 case and death data on PIs. Moreover, no states reported any granular data on PIs except for Hawaii [23]. As of October 16th, 2020, Hawaii’s Department of Health website reported “Native Hawaiians” and “Other Pacific Islanders” as distinct categories [31]. Disaggregating the data as such revealed a disparity between Other Pacific Islanders and Native Hawaiians, showing a higher rate of COVID-19 cases in Other Pacific Islanders than Native Hawaiians. Additionally, the CDC dataset does not report death counts less than 10 and the ACS dataset does not report population data for all races by state, both of which limit the potential to calculate and report SMRs. Furthermore, due to limited data, we do not report data on US territories, especially those with high PI populations such as American Samoa and Guam. Health disparities within US territories are poorly understood, and there is a corresponding lack of literature examining the impact of COVID-19 on these territories [32–34]. Future research must focus on the health of these US territories, especially as it pertains to PI health disparities.

With regard to the currently available data, the numbers of COVID-19 cases or deaths are likely underreported since this data were collected on Native Hawaiian or other Pacific Islanders as a single racial category, despite a majority of PIs (57%) reporting more than one race [11, 35]. Therefore, if the data were to take into account PIs of mixed race, rates of COVID-19 cases and deaths in PIs are likely higher than reported in this study. Accurate reporting of states with a greater proportion of COVID-19 cases or deaths is further complicated by Utah and Wyoming reporting non-mutually exclusive categories for race data. Eight states also reported COVID-19 cases or deaths for Pacific Islanders and Asians as a single pan-racial category, highlighting the long-standing issue of the lack of disaggregated data on PI and Asian populations.

Our findings illustrate the vital need and urgency for the medical community, public health organizations, and policymakers to recognize and address the disproportionate burden that COVID-19 is having on PIs and the distinct ethnic subgroups within this population. We urge prioritization of medical and public health outreach efforts in partnership with PI community-based and faith-based organizations to reduce and prevent COVID-19 in the states where PIs are most adversely affected. The monitoring and reporting of COVID-19 cases and deaths should be timely and consistent with PI data classification by distinct racial/ethnic subgroups (Native Hawaiian, Guamanian or Chamorro, Samoan, and Other Pacific Islander) as outlined by the OMB Directive-15 [7] and DHHS Data Collection Standards [8] for all US states and PI territories. Partnering with PI organizations who collect granular COVID-19 PI data, such as the Pacific Islander COVID-19 Response Team and UCLA Center for Health Policy Research, is vital to bridging this data gap in identifying needs and services for PIs.

In accordance with the DHHS Plan for Asian American, Native Hawaiian, and Pacific Islander Health, the health workforce lacks PI representation and must align with the needs of PI communities [36]. This effort necessitates national initiatives for PI health workforce employment and training programs. Community health partnerships are vital to building the PI health workforce across US states, PI territories, local county programs, PI community- and faith-based organizations, and schools. Groups with already established relationships and trust from PI communities can more effectively engage them in reducing COVID-19 spread and prevention, such as participation in health promotion programs, testing and contact tracing, and vaccinations [37]. These partnerships are also central to promoting culturally appropriate, in-language, health literate outreach efforts to ensure all PIs have access to needed health and social services. Additionally, these partnerships play an important role in developing information-sharing systems with local health officials, such as on COVID-19 data collection and reporting, to better understand the impacts of COVID-19 on PI communities. Taken together, these initiatives, along with increased national awareness and policy changes for PIs, are critical to mitigate the effects of historical suffering, health inequities, and COVID-19 facing this overlooked community in crisis.
